# SiO_2_@Benzothiazole‐Cl@Fc as an Efficient Heterogeneous Catalyst for the Synthesis of 1,3,5‐Trisubstituted Pyrazoles by A^3^ Coupling

**DOI:** 10.1002/open.202500024

**Published:** 2025-05-13

**Authors:** Fadhil Faez Sead, Vicky Jain, Roopashree R, Aditya Kashyap, Suman Saini, Girish Chandra Sharma, Pushpa Negi Bhakuni, Mosstafa Kazemi, Ramin Javahershenas

**Affiliations:** ^1^ Department of Dentistry College of Dentistry The Islamic University Najaf Iraq; ^2^ Department of Medical analysis Medical Laboratory Technique College The Islamic University of Al Diwaniyah Al Diwaniyah Iraq; ^3^ Department of Medical Analysis Medical Laboratory Technique College The Islamic University of Babylon Babylon Iraq; ^4^ Marwadi University Research Center Department of Chemistry Faculty of Science Marwadi University Rajkot Gujarat 360003 India; ^5^ Department of Chemistry and Biochemistry School of Sciences JAIN (Deemed to be University) Bangalore Karnataka India; ^6^ Centre for Research Impact & Outcome Chitkara University Institute of Engineering and Technology Chitkara University Rajpura Punjab 140401 India; ^7^ Department of Chemistry Chandigarh Engineering College Chandigarh Group of Colleges‐Jhanjeri Mohali Punjab 140307 India; ^8^ Department of Applied Sciences‐Chemistry NIMS Institute of Engineering & Technology NIMS University Rajasthan Jaipur India; ^9^ Department of Allied Science Graphic Era Hill University Bhimtal India; ^10^ Graphic Era Deemed to be University Dehradun Uttarakhand India; ^11^ Young Researchers and Elite Club Tehran Branch Islamic Azad University Tehran Iran; ^12^ Department of Organic Chemistry Faculty of Chemistry Urmia University Urmia Iran

**Keywords:** A3 coupling, ferrocene, heterogeneous catalyst, ionic liquid, pyrazole synthesis, silica nanospheres

## Abstract

This research introduces the preparation and analysis of a newly heterogeneous catalyst developed silica nanospheres supporting a ferrocene‐containing ionic liquid (IL) (SiO_2_@Benzothiazole‐Cl@Fc) for the A3 coupling reaction. The catalyst facilitates the efficient synthesis of 1,3,5‐trisubstituted pyrazoles from aromatic hydrazides, aldehydes, and aromatic alkynes. Incorporating ferrocene enhances the catalytic activity. Comprehensive characterization techniques, including NMR, Fourier transform infrared, transmission electron microscopy, energy‐dispersive X‐ray spectroscopy, and scanning electron microscopy, confirm the successful functionalization of silica nanospheres. The catalytic performance was evaluated under various reaction conditions, demonstrating high yields and selectivity for the desired pyrazole products. This work highlights the potential of ferrocene‐based ILs in green chemistry applications, providing a sustainable approach to synthesizing valuable heterocyclic compounds.

## Graphical abstract

This research introduces the preparation and analysis of a newly heterogeneous catalyst developed silica nanospheres supporting a ferrocene‐containing ionic liquid (SiO_2_@Benzothiazole‐Cl@Fc) for the A3 coupling reaction. The catalyst facilitates the efficient synthesis of 1,3,5‐trisubstituted pyrazoles from aromatic hydrazides, aldehydes, and aromatic alkynes.

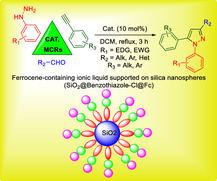

## 1 Introduction

Traditional methods for synthesizing these compounds often demand harsh conditions and prolonged reaction times, frequently leading to low yields. As a result, researchers continually seek more efficient and sustainable synthetic strategies. One promising approach is the one‐pot multicomponent reaction (MCR) paradigm, which facilitates the simultaneous condensation of multiple reagents to construct complex structures. This method enhances efficiency and minimizes by‐product formation, establishing itself as a valuable tool in organic synthesis.^[^ ^1^, ^2^, ^3^, ^4^, ^5^ ^]^

Developing efficient and sustainable catalytic methodologies remains a critical challenge system for organic transformations and is a cornerstone of modern synthetic chemistry. Among these, the design of heterogeneous catalysts has garnered significant attention due to their ease of recyclability, recovery, operational simplicity, reusability, and potential for minimizing waste. In this context, the innovative silica nanospheres supporting a ferrocene‐containing ionic liquid (IL) (SiO_2_@Benzothiazole‐Cl@Fc) emerge as a promising catalyst platform, strategic incorporation of ferrocene, a versatile metallocène, and the use of silica nanospheres provide unique opportunities for enhanced catalytic performance, improved substrate accessibility, and potential green chemistry applications.^[^ ^6^, ^7^, ^8^, ^9^ ^]^

Ferrocene, a well‐known organometallic compound, has been widely explored in catalysis due to its redox activity, thermal stability, and unique electronic properties. Integrating ferrocene into IL‐based systems has opened new avenues for designing multifunctional catalysts with enhanced activity and selectivity. In particular, silica nanospheres supporting a ferrocene‐containing IL (SiO_2_@Benzothiazole‐Cl@Fc) represent a promising class of materials for catalytic applications, combining the structural advantages of silica nanospheres with the catalytic potential of ferrocene and ILs.^[^ ^10^, ^11^, ^12^, ^13^, ^14^, ^15^ ^]^

The ILs have emerged as versatile materials in catalysis, offering unique properties such as tunable polarity, high thermal stability, and the ability to dissolve a wide range of substrates. Incorporating ILs onto solid supports, such as silica nanospheres, further enhances their catalytic performance by combining the advantages of homogeneous and heterogeneous catalysis. This hybrid approach enhances catalyst reusability and stability while simplifying product separation, contributing to a more environmentally friendly process. However, using ILs as homogeneous catalysts often suffers from catalyst recovery and reuse difficulties, leading to increased costs and environmental concerns.^[^ ^16^, ^17^, ^18^, ^19^, ^20^ ^]^

To address these limitations, the immobilization of ILs on solid supports has emerged as a promising strategy for developing heterogeneous catalysts. Silica nanospheres, in particular, have been widely used as supports due to their high surface area, stability, and ease of functionalization. Incorporating ferrocene (Fc) units into ILs has also been shown to enhance their catalytic activity due to the ability of Fc to participate in redox reactions and facilitate the formation of reactive intermediates.^[^ ^21^, ^22^, ^23^, ^24^, ^25^ ^]^

Pyrazoles, a prominent class of nitrogen‐containing heterocycles, have garnered significant attention due to their diverse biological and pharmaceutical applications. The A3 coupling reaction, which enables the one‐pot synthesis of 1,3,5‐trisubstituted pyrazoles, represents an elegant approach to constructing these valuable molecular scaffolds. The A3 coupling reaction, involving the three‐component condensation of aldehydes, alkynes, and amines, is a powerful tool for synthesizing nitrogen‐containing heterocycles. This reaction has been extensively studied due to its atom economy, operational simplicity, and ability to generate structurally diverse products. However, the extension of this methodology to the synthesis of 1,3,5‐trisubstituted pyrazoles, a class of compounds with significant pharmaceutical and biological importance, remains underexplored. Using aromatic hydrazides as nitrogen sources in A3 coupling reactions offers a direct and efficient route to these valuable heterocycles. However, it requires the development of mild and efficient catalytic systems to achieve high yields and selectivity.^[^ ^26^, ^27^, ^28^, ^29^, ^30^ ^]^

**Figure 1:**
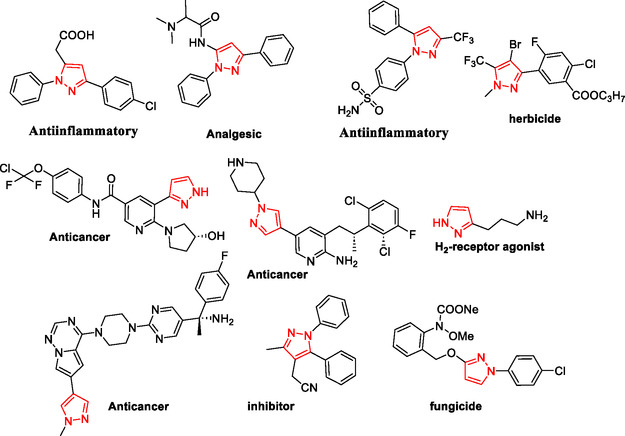
Some bioactive compounds contain structures containing substituted pyrazoles derivatives.

Pyrazoles and their derivatives represent an important class of heterocyclic compounds used extensively in the pharmaceutical and agrochemical industries.^[^ ^17^, ^31^, ^32^, ^33^, ^34^, ^35^, ^36^, ^37^, ^38^, ^39^ ^]^ Pyrazoles have continued to attract considerable attention due to the broad range of biological activities,^[^ ^40^, ^41^, ^42^, ^43^, ^44^ ^]^ including analgesia,^[^ ^45^, ^46^ ^]^ antibacterial,^[^ ^47^, ^48^ ^]^ antidepressant,^[^ ^46^ ^]^ anti‐inflammatory,^[^ ^47^, ^48^ ^]^ antimicrobial,^[^ ^47^, ^48^, ^49^ ^]^ antiobesity,^[^ ^50^, ^51^ ^]^ antiviral,^[^ ^52^ ^]^ appetite suppressant,^[^ ^53^, ^54^ ^]^ cholesterol‐lowering,^[^ ^55^ ^]^ hypoglycemic,^[^ ^56^, ^57^ ^]^ antihypertensive,^[^ ^58^ ^]^ and anticancer,^[^ ^59^, ^60^, ^61^, ^62^ ^]^ as well as the HIV‐1 reverse transcriptase inhibitor,^[^ ^63^ ^]^ inhibitor,^[^ ^48^ ^]^ herbicide,^[^ ^64^ ^]^ and fungicide activities^[^ ^65^, ^66^, ^67^ ^]^ (**Figure** **1**).

A3 coupling reactions, which involve the one‐pot condensation of aldehydes, amines, and alkynes, represent a powerful and highly efficient strategy for constructing complex molecular architectures, particularly in the synthesis of 1,3,5‐trisubstituted pyrazoles. The significance of A3 coupling lies in its ability to generate diversity in products while minimizing the need for extensive purification steps, thereby enhancing overall reaction efficiency and yield. The introduction of SiO_2_@Benzothiazole‐Cl@Fc significantly enhances the catalytic performance of A3 coupling reactions, offering increased recyclability and ease of separation from the reaction mixture, which is critical for practical applications in synthetic organic chemistry. This enhancement piques interest in the potential of A3 coupling reactions and SiO_2_@Benzothiazole‐Cl@Fc in sustainable and efficient chemical processes.^[^ ^68^, ^69^, ^70^, ^71^, ^72^ ^]^

In this study, we report the synthesis and application of a silica nanospheres supporting a ferrocene‐containing IL (SiO_2_@Benzothiazole‐Cl@Fc) as a heterogeneous catalyst for the A3 coupling of aromatic hydrazides, aldehydes, and aromatic alkynes. The catalyst demonstrates excellent activity and selectivity under mild reaction conditions, providing a green and efficient approach to synthesizing 1,3,5‐trisubstituted pyrazoles. The structural and catalytic properties of the material were thoroughly characterized, and its reusability and stability were evaluated, highlighting its potential for practical applications in organic synthesis.

## 2 Results and Discussion

The development of SiO_2_@Benzothiazole‐Cl@Fc as a heterogeneous catalyst represents a significant advancement in organic synthesis, particularly for the efficient production of 1,3,5‐trisubstituted pyrazoles. Combining the unique properties of ILs with silica nanospheres’ stability and functionalization capabilities, this catalyst is designed to facilitate a MCR, a process involving the simultaneous transformation of multiple starting materials, under mild conditions.

The integration of ferrocene enhances both the catalytic activity and the recovery process, allowing for straightforward reuse without compromising performance. The study, conducted with thoroughness and precision, elucidates the synthesis and characterization processes and emphasizes the catalyst's application in the A3 coupling reaction. Our findings, a testament to the reliability of our study, indicate that SiO_2_@Benzothiazole‐Cl@Fc streamlines the reaction sequence and contributes to greater yields and selectivity for the desired products. This approach paves the way for greener synthetic methodologies while addressing the challenges in organic transformations.

**Table** **1** presents the optimization of the synthesis of a substituted 1,3,5‐trisubstituted pyrazoles moiety (4a) from benzaldehyde (**1a**), phenylhydrazine (**2a**), and phenylacetylene (**3a**), catalyzed by SiO_2_@Benzothiazole‐Cl@Fc. The key parameters investigated were the amount of catalyst, solvent, temperature, and reaction time.

**Table 1:** Optimization of the synthesis of substituted 1,3,5‐trisubstituted pyrazoles moiety.

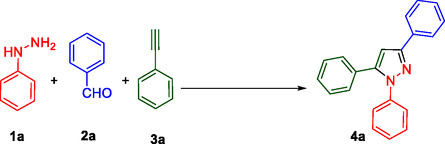					
Entry	Catalyst [mg] SiO_2_@Benzothiazole‐Cl@Fc	Solvent	T [°C]	Time [h]a)	Yield [%]b)
1	–	CH_2_Cl_2_	25	12	19
2	–	CH_2_Cl_2_	Reflux	12	28
3	–	CH_2_Cl_2_	Reflux	24	47
4	5	CH_2_Cl_2_	Reflux	1	65
5	10	CH_2_Cl_2_	Reflux	1	72
6	10	CH_2_Cl_2_	Reflux	2	86
7	**10**	**CH**_**2**_ **Cl**_**2**_	Reflux	**3**	**95**
8	10	CH_2_Cl_2_	Reflux	4	93
9	15	CH_2_Cl_2_	Reflux	3	92
10	10	Ethanol	Reflux	3	89
11	10	Ethanol: water (1:1)	Reflux	3	85
12	10	H_2_O	Reflux	3	81
13	10	CH_3_Cl	Reflux	3	74
14	10	CH_3_CN	Reflux	3	79

a) Reaction conditions: benzaldehyde 1a (0.5 mmol), phenylhydrazine 2a (0.5 mmol), phenylacetylene 3a (0.6 mmol), and in solvent (2 mL).

b) Isolated yield

The initial experiments (entries 1 and 2) explored the reaction without a catalyst at 25 °C and reflux in CH_2_Cl_2_, yielding only 19% and 28% of the product, respectively. This stark contrast highlights the indispensable role of the catalyst. Increasing the catalyst amount to 5 mg (entry 3) and maintaining reflux in CH_2_Cl_2_ for 24 h significantly improved the yield to 47%, instilling confidence in the catalyst's effectiveness. Further optimization of the reaction time with 5 mg of catalyst showed that a shorter reaction time of 1 h could achieve a 65% yield (entry 4).

Increasing the catalyst loading to 10 mg significantly enhanced the reaction rate. With 10 mg of catalyst in CH_2_Cl_2_ under reflux, the reaction reached 72% yield in just 1 h (entry 5). Extending the reaction time to 2 and 3 h increased the yield to 86% (entry 6) and 95% (entry 7), respectively. A longer reaction time of 4 h with 10 mg of catalyst did not improve the yield, slightly decreasing it to 93% (entry 8). This suggests that 3 h is the optimal reaction time with 10 mg of catalyst. Increasing the catalyst amount to 15 mg (entry 9) did not improve the yield over the optimized conditions with 10 mg of catalyst and 3 h of reaction time.

The solvent also played a crucial role. When CH_2_Cl_2_ was replaced with ethanol (entry 10), the yield decreased to 89%. Using a mixture of ethanol and water (1:1) or water alone (entries 11 and 12) further reduced the yield to 85% and 81%, respectively. Other solvents like CH_3_Cl and CH_3_CN (entries 13 and 14) also resulted in lower yields (74% and 79%, respectively).

CH_2_Cl_2_ is the best solvent among those tested. The reasons for this are likely multifaceted, as CH_2_Cl_2_ is a suitable solvent for organic compounds, likely providing better solubility for the reactants and the catalyst than more polar solvents like ethanol and water. This enhanced solubility facilitates better interaction between the reactants and the catalyst, leading to a higher reaction rate and yield. CH_2_Cl_2_ is relatively inert under the reaction conditions, minimizing potential side reactions or interactions with the catalyst. In contrast, protic solvents like ethanol and water could interact with the catalyst or participate in unwanted side reactions, lowering the yield. The reflux temperature of CH_2_Cl_2_ is suitable for this reaction, providing sufficient energy for the reaction to proceed at a reasonable rate without being so high as to cause decomposition of the reactants or products.

In summary, the optimization study demonstrates that the combination of 10 mg of SiO_2_@Benzothiazole‐Cl@Fc catalyst, CH_2_Cl_2_ as a solvent, reflux temperature, and a reaction time of 3 h provides the highest yield (95%) for the synthesis of the substituted 1,3,5‐trisubstituted pyrazoles moiety.

### 2.1 Typical Procedure for Synthesizing the SiO_2_@Benzothiazole‐Cl@Fc Nanocatalyst

**Scheme 1:**
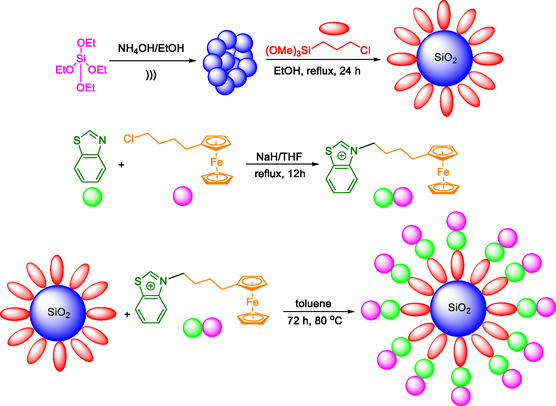
Preparation steps for synthesis of SiO_2_@Benzothiazole‐Cl@Fc nanocatalyst.

The synthetic approach outlined in **Scheme** **1** offers a straightforward and efficient route to preparing SiO_2_@Benzothiazole‐Cl@Fc nanocatalyst. Using reflux conditions and appropriate solvents ensures high yields and purity of the final product. The multistep process allows for precise control over the structure and properties of the nanocatalyst and ensures its reliability and consistency. Incorporating ferrocene units provides the catalyst with redox activity, while the thiazole ligand acts as a chelating agent to stabilize the metal center. This combination of features makes the SiO_2_@Benzothiazole‐Cl@Fc nanocatalyst a promising candidate for various catalytic applications.

**Scheme 2:**
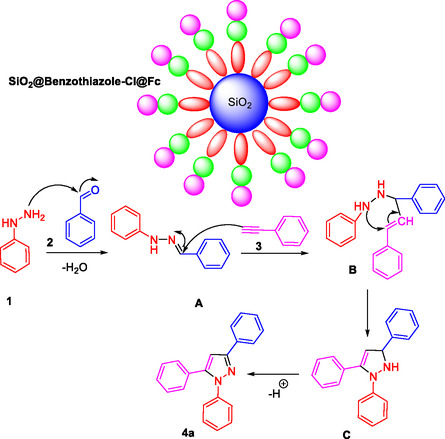
A plausible mechanism for the synthesis of polyfunctionalized 1,3,5‐trisubstituted pyrazoles derivatives.

Monodisperse silica nanospheres (SiO_2_) were synthesized using Stöber's method, a widely recognized and efficient technique that involves the hydrolysis of tetraethyl orthosilicate (TEOS) with ammonium hydroxide in ethanol. This method was chosen for its ability to produce uniform and monodisperse silica nanoparticles. For the synthesis of SiO_2_ nanoparticles, first, TEOS is hydrolyzed in an ammonia/ethanol solution to form SiO_2_ nanoparticles. Secondly, for the functionalization of SiO_2_ nanoparticles, the SiO_2_ nanoparticles are functionalized with (3‐chloropropyl)trimethoxysilane (CPTMS) in ethanol under reflux conditions. Then, for the synthesis of benzothiazole‐Cl ligand, benzo[d]thiazole and chlorobutyl ferrocene are reacted in the presence of NaH/THF under reflux conditions to form the 1‐*N*‐Ferrocenbutyl thiazole. Finally, for immobilization of benzothiazole‐Cl ligand on SiO_2_ Nanoparticles, the functionalized SiO_2_ nanoparticles are reacted with the 1‐*N*‐Ferrocenbutyl benzothiazole in toluene at 80 °C for 72 h to form the ferrocene‐containing IL supported on silica **Scheme** **2**.

The proposed mechanism for the synthesis of polyfunctionalized 1,3,5‐trisubstituted pyrazole derivatives, as catalyzed by the SiO_2_@Benzothiazole‐Cl@Fc nanocatalyst, unfolds through a series of well‐defined steps that illustrate the catalytic role of this hybrid material in facilitating the reaction. The reaction begins with condensing hydrazine derivative 1 and an aldehyde compound 2. The catalyst's surface, composed of silica (SiO_2_) functionalized with benzothiazole and ferrocene moieties, provides an active environment that enhances the electrophilicity of the aldehyde carbonyl group, thereby promoting nucleophilic attack by the hydrazine nitrogen.

In this first stage, water is eliminated as a by‐product, forming intermediate A, characterized by a hydrazone linkage. The catalyst's surface likely stabilizes this intermediate through interactions between its functional groups and the substrate molecules, thus lowering activation energy and increasing reaction efficiency. Subsequently, intermediate A undergoes intramolecular cyclization facilitated by the nucleophilic attack of another nitrogen atom on an adjacent electrophilic site within the molecule. This step forms intermediate B, where a new C—N bond is established, creating a heterocyclic ring structure characteristic of pyrazoles.

The SiO_2_@Benzothiazole‐Cl@Fc catalyst plays a crucial role by providing a confined microenvironment that orients reactants optimally and stabilizes transition states through electronic interactions derived from its ferrocene and benzothiazole functionalities. Following ring closure, proton transfer events occur to yield intermediate C. These proton shifts are essential for tautomerization and stabilization of the newly formed heterocycle. Finally, deprotonation leads to the aromatization of the pyrazole ring system, resulting in the formation of product 4a.

Throughout this mechanism, the catalyst not only accelerates each step but also enhances selectivity toward the desired trisubstituted pyrazole derivatives by minimizing side reactions. The silica core provides structural support and a high surface area for effective dispersion of active sites, while benzothiazole groups may participate in hydrogen bonding or π–π interactions with substrates. The ferrocene units contribute redox‐active properties that could facilitate electron transfer processes during bond formation and cleavage.

The SiO_2_@Benzothiazole‐Cl@Fc nanocatalyst is a multifunctional platform that synergistically combines physical adsorption capacity with chemical activation capabilities. This dual role ensures the efficient conversion of starting materials into highly substituted pyrazoles under mild conditions with improved yields and selectivity. The detailed mechanistic insights underscore how the rational design of hybrid catalysts can enable complex organic transformations by orchestrating substrate activation, intermediate stabilization, and product formation within a single catalytic framework.

To investigate the electronic and spatial effects of substituent groups on reactants, we performed a series of reactions with alkynes, substituted phenylhydrazines, and various aldehydes to synthesize 1,3,5‐trisubstituted pyrazole derivatives. The reactions were conducted under reflux using 10 mg of SiO_2_@Benzothiazole‐Cl@Fc as the catalyst. The results summarized in **Table** **2** indicate that aromatic aldehydes with electron‐withdrawing substituents demonstrated significantly faster reaction rates and higher yields in producing the desired 1,3,5‐trisubstituted pyrazole derivatives.

**Table 2:** SiO_2_@Benzothiazole‐Cl@Fc nanocatalyst‐catalyzed synthesis of 1,3,5‐trisubstituted pyrazole derivatives.

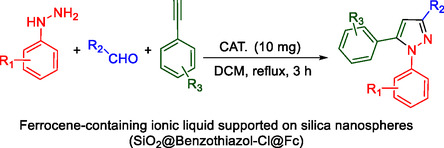			
Ferrocene‐containing IL supported on silica nanospheres (SiO_2_@Benzothiazol‐Cl@Fc)			
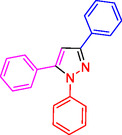	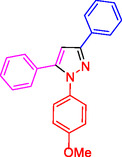	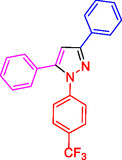	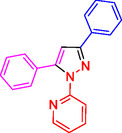
**4a, 95%**	**4b, 94%**	**4c, 96%** a)	**4d, 86%** b)
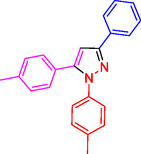	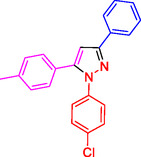	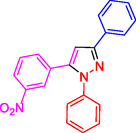	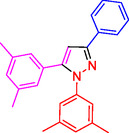
**4e, 89%**	**4f, 92%**	**4g, 86%**	**4h, 93%**
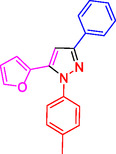	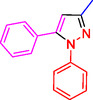	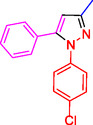	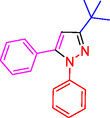
**4i, 85%**	**4j, 91%,**	**4 k, 87%**	**4L, 84%**
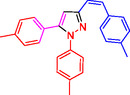	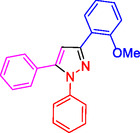	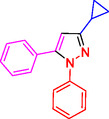	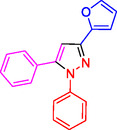
**4m, 90%**	**4n, 91%**	**4o, 90%**	**4p, 91%**
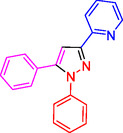	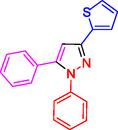		
**4q, 91%**	**4r, 90%**

a) Reaction conditions: benzaldehyde 1a (0.5 mmol), phenylhydrazine 2a (0.5 mmol), phenylacetylene 3a (0.6 mmol), and catalyst in solvent (2 ml), under reflux and ultrasound conditions for 3 h.

b) Isolated yield

### 2.2 Characterization of SiO_2_@Benzothiazole‐Cl@Fc Nanocatalyst

**Figure 2:**
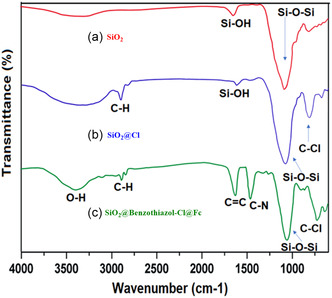
FTIR spectrum of a) SiO_2_, b) SiO_2_@Cl, and c) SiO_2_@Benzothiazole‐Cl@Fc nanocatalyst.

The FTIR spectra of SiO_2_, SiO_2_@Cl, and SiO_2_@Benzothiazole‐Cl@Fc nanocatalysts provide insightful information regarding the structural and functional group modifications occurring during the catalyst synthesis. **Figure** **2** compares the Fourier transform infrared (FTIR) spectra of SiO_2_, SiO_2_@Cl, and SiO_2_@Benzothiazole‐Cl@Fc Nanocatalysts. The FTIR spectra of the three nanocatalysts were recorded in the wavenumber range of 4000–500 cm^−1^.

The spectrum of pure SiO_2_ (curve a) exhibits characteristic absorption bands indicative of its silica framework. Notably, a broad peak around 3400 cm^−^^1^ corresponds to the stretching vibrations of surface hydroxyl groups (Si—OH). In comparison, a strong and sharp band near 1100 cm^−^^1^ is attributed to the asymmetric stretching vibrations of siloxane bonds (Si—O—Si). These features confirm the presence of silanol groups and the silica network in the pristine material.

Notably, changes occur upon functionalization with chlorine‐containing moieties, as seen in the spectrum of SiO_2_@Cl (curve b). The broad Si—OH band remains present but appears slightly diminished, suggesting partial consumption or modification of surface hydroxyl groups during chlorination. Additionally, new absorption bands emerge around 2900 cm^−^^1^ corresponding to C—H stretching vibrations, indicating successful grafting of organic chlorinated groups onto the silica surface. Notably, a peak near 650 cm^−^^1^ is observed, which can be assigned to C—Cl stretching vibrations, confirming the presence of chlorine functionalities on the modified silica. The Si—O—Si band retention near 1100 cm^−^^1^ indicates that the silica framework remains intact after functionalization.

Further modification to obtain SiO_2_@Benzothiazole‐Cl@Fc nanocatalyst (curve c) introduces additional spectral features incorporating benzothiazole and ferrocene moieties. The O—H stretching band around 3400 cm^−^^1^ becomes broader and more pronounced compared to curve b, suggesting either an increase in hydrogen bonding or residual hydroxyl groups possibly associated with ligand coordination. The C—H stretching vibrations remain evident near 2900 cm^−^^1^, consistent with organic ligand presence. New peaks appear in the region between 1600 and 1400 cm^−^^1^, characteristic of C=C and C—N stretching vibrations; these bands confirm successful attachment of benzothiazole rings containing nitrogen heteroatoms onto the silica surface. The C—Cl peak at ≈650 cm^−^^1^ persists but with slight intensity variation, indicating that some chlorine atoms remain after further functionalization steps. Moreover, multiple Si—O—Si bands are still present near 1100 cm^−^^1^ and below 1000 cm^−^^1^, demonstrating that the silica network remains structurally preserved throughout all modification stages.

Comparative analysis of these spectra reveals a clear progression from bare silica to a complex hybrid nanocatalyst system. Initially, pure SiO_2_ shows typical silanol and siloxane features without organic signatures. Chlorination introduces C—H and C—Cl functionalities while partially reducing surface hydroxyls but preserving the silica matrix. Subsequent conjugation with benzothiazole‐ferrocene ligands adds heteroatom‐containing functional groups such as C=N and aromatic C=C bonds alongside retained chlorine sites, thereby enriching chemical complexity and potential catalytic activity.

This FTIR analysis confirms each step of catalyst preparation by identifying specific vibrational modes corresponding to introduced functional groups while verifying that the fundamental silica scaffold remains intact. The successful synthesis of the multifunctional nanocatalyst, with the effective immobilization of benzothiazole‐ferrocene units onto chlorinated silica, suggests a significant achievement in the field of nanocatalyst design.

**Figure 3:**
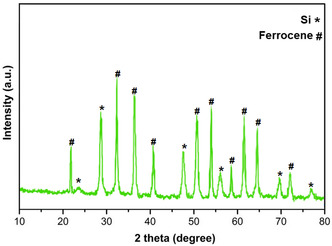
XRD analysis of SiO_2_@Benzothiazole‐Cl@Fc nanocatalyst.

The X‐ray diffraction (XRD) analysis of the SiO_2_@Benzothiazole‐Cl@Fc nanocatalyst provides critical insights into its crystalline structure and phase composition. The diffraction pattern, depicted in **Figure** **3**, reveals distinct peaks corresponding to the crystalline phases in the material. Notably, the peaks marked with asterisks (*) are attributed to the silica (SiO_2_) framework, while those denoted by hash symbols (#) are associated with ferrocene.

The characteristic peaks for SiO_2_ appear prominently at specific 2θ angles, indicating a well‐defined crystalline structure typical of amorphous silica. These peaks reflect the long‐range order inherent in the silica matrix, essential for maintaining structural integrity during catalytic applications. Sharp and intense diffraction peaks suggest a high degree of crystallinity in the silica component, confirming that the synthesis process effectively preserved its structural properties.

In contrast, the ferrocene‐related peaks are less intense and more diffuse than silica. This observation indicates that ferrocene may be present within the composite material in a less ordered or amorphous state. The less intense and more diffuse peaks suggest a lower degree of crystallinity in the ferrocene component, which could potentially affect its electron transfer properties and, consequently, its catalytic activity. Incorporating ferrocene into the silica matrix is crucial as it introduces redox‐active sites that can enhance catalytic activity through electron transfer. The relative intensities of these peaks suggest that while ferrocene is successfully integrated into the catalyst framework, its overall contribution to crystallinity is lower than that of silica.

Comparative analysis of both components reveals a synergistic relationship. The robust framework provided by SiO_2_ supports the functionalization with benzothiazole and ferrocene and ensures stability under operational conditions, which in this context refer to the conditions typically encountered in catalytic reactions. The presence of well‐defined silica peaks alongside less pronounced ferrocene signals implies that while ferrocene contributes to the catalytic functionality, it does so without significantly disrupting the underlying silica structure.

Moreover, this XRD analysis underscores the successful synthesis of a hybrid nanocatalyst where both inorganic and organic components coexist harmoniously. The retention of silica's crystalline features alongside the integration of ferrocene highlights a practical approach to developing multifunctional catalysts that leverage structural stability and enhanced reactivity, inspiring further research and development in this area.

XRD data elucidates key aspects of the SiO_2_@Benzothiazole‐Cl@Fc nanocatalyst's composition and structure. The clear differentiation between silica and ferrocene signals confirms successful synthesis and underscores their complementary roles in enhancing catalytic performance.

**Figure 4:**
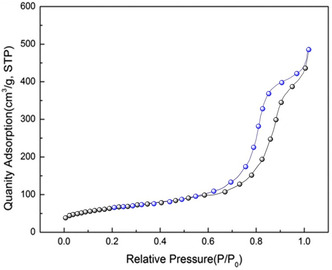
The N_2_ adsorption‐desorption isotherm of SiO_2_@Benzothiazole‐Cl@Fc nanocatalyst.

The nitrogen (N_2_) adsorption‐desorption isotherm of the SiO_2_@Benzothiazole‐Cl@Fc nanocatalyst, illustrated in **Figure** **4**, provides critical insights into this hybrid material's porous characteristics and surface area. The graph plots the quantity of nitrogen adsorbed (in cm^3^ g^−1^ STP) against the relative pressure (P/P_0_), revealing a clear trend that characterizes the material's porosity.

At low relative pressures, specifically in the range of 0.0 to ≈0.3 P/P_0_, nitrogen adsorption gradually increases, indicative of monolayer adsorption on the surface of the silica framework. This initial uptake suggests high accessibility to the surface area, which is essential for catalytic applications where reactants must interact with active sites on the catalyst.

As the relative pressure increases beyond 0.3 P/P_0_, a more pronounced increase in adsorption occurs, culminating in a significant rise around 0.5 P/P_0_. According to the IUPAC classification, this behavior is characteristic of Type IV isotherms, which typically indicate mesoporous materials. The steep increase in adsorption at this stage suggests that capillary condensation is taking place within mesopores, allowing additional nitrogen molecules to be accommodated within these voids.

The desorption branch of the isotherm closely follows the adsorption curve but shows a slight hysteresis loop between 0.4 and 0.8 P/P_0_. Hysteresis, in this context, refers to the phenomenon where not all adsorbed nitrogen is released upon depressurization. This indicates potential structural features such as pore connectivity, which is the degree to which pores are interconnected, or narrow pore throats that can trap gas molecules. Hysteresis is often associated with materials that exhibit complex pore structures and can imply that some pores are not fully accessible or are partially blocked.

At higher relative pressures approaching unity (1.0 P/P_0_), both adsorption and desorption curves converge, suggesting that saturation has been reached and confirming that the most available pore volume has been utilized for nitrogen storage. The maximum quantity adsorbed approaches ≈600 cm^3^ g^−1^ STP, indicating a substantial surface area that is truly impressive and can be advantageous for catalytic processes by providing numerous active sites.

Comparative analysis of these features reveals that SiO_2_@Benzothiazole‐Cl@Fc exhibits favorable porous characteristics that are highly conducive to enhancing its catalytic performance. The microporosity indicated by the type IV isotherm signifies that this nanocatalyst can facilitate the efficient diffusion of reactants and products during chemical reactions, thereby potentially increasing reaction rates.

In conclusion, the N_2_ adsorption‐desorption isotherm analysis elucidates key aspects of the SiO_2_@Benzothiazole‐Cl@Fc nanocatalyst's structure and porosity. The findings suggest that this hybrid material possesses an extensive surface area and well‐defined mesoporosity, critical factors contributing to its efficacy as a catalyst. The potential for future studies to further explore how these structural attributes correlate with specific catalytic activities and efficiencies in various chemical transformations is promising and should be eagerly anticipated.

**Figure 5:**
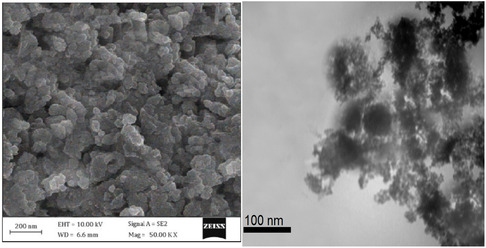
SEM and TEM images of SiO_2_@Benzothiazole‐Cl@Fc nanocatalyst.

The scanning electron microscopy (SEM) and transmission electron microscopy (TEM) images presented in **Figure** **5** provide detailed insights into the morphological and structural characteristics of the SiO_2_@Benzothiazole‐Cl@Fc nanocatalyst. The left panel illustrates the SEM image, which reveals a granular morphology characterized by agglomerated particles with an average size of hundreds of nanometers. The surface appears rough and uneven, indicative of a high degree of porosity that is often advantageous for catalytic applications, suggesting a promising potential for this nanocatalyst in practical use.

In contrast, the TEM image on the right side offers a closer examination at a higher resolution, showcasing individual nanoparticles with a more defined structure. The scale bar in this image indicates that the primary particles are ≈100 nm or smaller, suggesting that despite the apparent agglomeration observed in SEM, the individual units of the nanocatalyst are relatively small. This fine structure is critical as it can significantly influence catalytic activity; smaller nanoparticles typically exhibit higher surface area‐to‐volume ratios, leading to enhanced reactivity.

When comparing both imaging techniques, it becomes evident that while SEM provides valuable information regarding overall morphology and particle distribution, TEM allows for a more nuanced understanding of particle size and internal structure. Combining these two imaging methods paints a comprehensive picture of the nanocatalyst's characteristics. The SEM results indicate potential aggregation issues, where the particles tend to clump together, that could affect performance by limiting effective surface area. This limitation could reduce the number of active sites available for the catalytic reaction, thereby decreasing the efficiency of the catalyst. In contrast, TEM highlights that these aggregates consist of smaller nanoparticles that may retain significant catalytic properties.

Moreover, the presence of benzothiazole and ferrocene (Fc) moieties within this composite structure suggests functionalization to improve catalytic efficiency. These organic components play a crucial role in enhancing the nanocatalyst's potential by contributing to specific interactions with substrates during catalytic processes. Integrating such functionalities can enhance electron transfer capabilities and stabilize reactive intermediates, ultimately improving catalytic performance.

The analysis of SEM and TEM images reveals crucial details about the SiO_2_@Benzothiazole‐Cl@Fc nanocatalyst's morphology and structural integrity. While SEM emphasizes particle agglomeration and surface characteristics, TEM elucidates finer structural features critical for understanding catalytic behavior. Together, these imaging techniques underscore the importance of morphology and nanoscale dimensions in optimizing catalyst design for enhanced activity in chemical reactions. It is clear that future studies should focus on correlating these structural insights with actual catalytic performance to fully exploit the advantages of such nanocomposite materials, highlighting the need for further research in this area.

**Figure 6:**
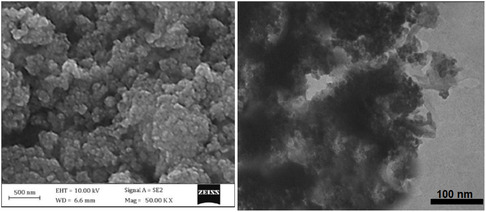
SEM and TEM images of SiO_2_ NPs.

The images presented in **Figure** **6** depict SEM and TEM analyses of silicon dioxide nanoparticles (SiO_2_ NPs), providing complementary insights into their morphological and structural properties. The SEM image on the left reveals a granular morphology characterized by agglomerated particles, with a scale bar indicating a size of 500 nm. This suggests that the SiO_2_ NPs form clusters, which may influence their surface area and reactivity in various applications. The rough and uneven surface texture observed in the SEM image indicates a high porosity, potentially enhancing the material's utility as a catalyst or adsorbent.

In contrast, the TEM image on the right offers a higher resolution view of the SiO_2_ NPs, with a scale bar of 100 nm. This image reveals finer details about individual nanoparticles, showing that they are significantly smaller than those observed in the SEM image. The TEM analysis indicates that while some aggregation is present, many particles maintain dimensions well below 100 nm, which is advantageous for applications requiring high surface area and reactivity.

Comparing both imaging techniques highlights the strengths of each method: SEM provides an overview of particle distribution and morphology at a larger scale, while TEM allows for a detailed examination of individual nanoparticle structures. The apparent discrepancy in particle size between SEM and TEM can be attributed to aggregation effects; despite forming larger clusters observable by SEM, many individual nanoparticles remain small enough to exhibit enhanced catalytic properties.

These findings underscore the importance of utilizing both SEM and TEM to comprehensively understand SiO_2_ NPs’ characteristics. The combination of insights from both techniques suggests that while agglomeration may pose challenges for specific applications, the intrinsic small size of individual SiO_2_ particles could still confer significant advantages in catalytic efficiency and material performance. Future research should further explore these aspects to optimize SiO_2_ NP applications across various fields.

**Figure 7:**
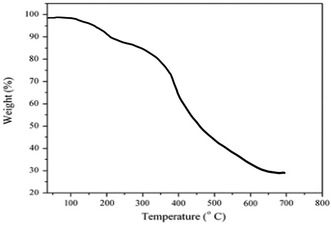
TGA analysis of SiO_2_@Benzothiazole‐Cl@Fc nanocatalystat.

The SiO_2_@Benzothiazole‐Cl@Fc nanocatalyst, a hybrid nanocatalyst composed of an inorganic silica core and organic surface modifications, is the subject of this study. The thermogravimetric analysis (TGA) curve of this nanocatalyst, as depicted in **Figure** **7**, provides critical insights into the material's thermal stability and composition. The TGA profile reveals a gradual weight loss starting from room temperature up to ≈700 °C. Initially, the sample maintains close to 100% of its original weight until around 100 °C, indicating a minimal loss in this range, which can be attributed primarily to the evaporation of physically adsorbed moisture or volatile impurities on the catalyst surface.

After 100 °C, a steady decrease in weight is observed, becoming more pronounced between 200 °C and 500 °C. This significant weight loss phase likely corresponds to the decomposition of organic functional groups attached to the silica support, including the benzothiazole moiety and ferrocene‐containing IL components. The continuous decline in weight over this temperature range suggests that these organic functionalities are thermally labile and degrade progressively rather than abruptly. By ≈700 °C, the residual mass stabilizes at around 30%, indicative of the inorganic silica framework's robustness and resistance to thermal degradation. This gradual weight loss pattern provides reassurance about the nanocatalyst's stability.

This TGA behavior aligns with expectations for hybrid nanocatalysts composed of an inorganic core and organic surface modifications. The high thermal stability of the silica core ensures structural integrity under elevated temperatures, while the organic layers contribute to catalytic activity but exhibit limited thermal endurance. The gradual decomposition pattern also implies strong covalent or ionic bonding between organic groups and the silica surface, as rapid or early detachment would manifest as sharp weight loss steps at lower temperatures.

When compared to similar catalysts in the literature, the SiO_2_@Benzothiazole‐Cl@Fc nanocatalyst stands out for its balanced combination of durability and functional complexity. This thermal profile confirms that while operating conditions should ideally remain below 200–300 °C to preserve catalytic sites fully, the catalyst can withstand moderate heating without complete structural collapse. This unique balance is a testament to the meticulous design and engineering that went into creating this nanocatalyst.

The TGA analysis underscores that the SiO_2_@Benzothiazole‐Cl@Fc nanocatalyst possesses excellent thermal stability attributable to its silica core, while its organic functional groups decompose gradually over a broad temperature range. This characteristic is crucial for practical applications where controlled heating is involved. It highlights the catalyst's suitability for reactions conducted under mild to moderate thermal conditions without significant loss of active sites, opening up exciting possibilities for its use in various catalytic processes.

**Figure 8:**
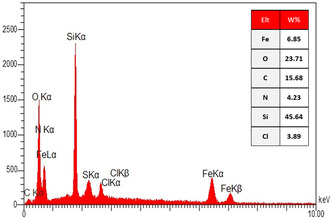
EDX analysis of SiO_2_@Benzothiazole‐Cl@Fc nanocatalystat.

**Figure** **8** presents the energy‐dispersive X‐ray spectroscopy (EDX) analysis of the SiO_2_@Benzothiazole‐Cl@Fc nanocatalyst, providing crucial insights into its elemental composition. The EDX spectrum illustrates distinct peaks corresponding to various elements, with the intensity of these peaks indicative of their respective weight percentages in the sample.

The quantitative data reveals a significant finding‐silicon (Si) is the most abundant element, constituting ≈45.64% of the total weight. This high proportion, which aligns with the expected presence of silica (SiO_2_), a foundational support material for the catalyst, underscores the importance of silicon in the nanocatalyst. Following silicon, oxygen (O) is also prevalent at 23.71%, reflecting its role in forming silicate structures and possibly participating in catalytic processes through surface interactions.

Carbon (C) accounts for 15.68%, suggesting a significant contribution from organic components within the benzothiazole‐Cl structure integrated into the catalyst framework. Nitrogen (N) at 4.23% indicates that functional groups associated with benzothiazole are effectively incorporated, potentially enhancing catalytic activity through specific interactions with substrates.

Iron (Fe), present at 6.85%, suggests that iron‐based species may play a pivotal role in facilitating catalytic reactions, likely through redox processes or as active sites for substrate binding and transformation. Additionally, chlorine (Cl) appears at 3.89%, which may indicate that chlorinated functionalities contribute to the electronic properties of the catalyst, thereby influencing its reactivity.

Comparing these results with typical compositions found in similar nanocatalysts highlights several important aspects. First, the significant presence of silicon and oxygen is consistent with silica‐supported catalysts known for their stability and effectiveness in various chemical reactions. Incorporating organic moieties such as benzothiazole enhances solubility and introduces specific reactive sites that can interact favorably with target molecules.

Moreover, the presence of iron as a transition metal is particularly exciting. It suggests potential applications in catalysis involving oxidation or reduction reactions where iron can facilitate electron transfer processes. This is further supported by the relatively moderate amount of nitrogen and chlorine, which may enhance selectivity and efficiency in specific catalytic pathways.

The EDX analysis provides a comprehensive view of the elemental composition of the SiO_2_@Benzothiazole‐Cl@Fc nanocatalyst. This thorough analysis reveals a well‐balanced integration of inorganic and organic components essential for catalytic performance. The synergistic effects arising from this composition will likely enhance both stability and reactivity, positioning this nanocatalyst as a promising candidate for further exploration in catalytic applications across diverse chemical transformations.

**Figure 9:**
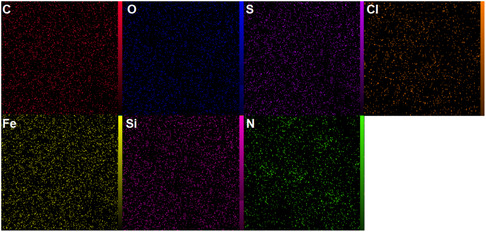
Elemental mapping of the SiO_2_@Benzothiazole‐Cl@Fc nanocatalystat.

The elemental mapping analysis of the SiO_2_@Benzothiazole‐Cl@Fc nanocatalyst, as illustrated in **Figure** **9**, provides a comprehensive visualization of the distribution of key elements within the composite material. Each panel in the figure corresponds to a specific element: carbon (C), oxygen (O), sulfur (S), chlorine (Cl), iron (Fe), silicon (Si), and nitrogen (N). This elemental mapping effectively highlights the spatial arrangement and relative abundance of these elements, which are critical for understanding the structural and functional properties of the nanocatalyst.

The carbon mapping reveals a significant presence throughout the sample, indicating that benzothiazole functionalities are well‐distributed within the silica matrix. This uniform distribution is essential for ensuring that the catalytic sites are readily accessible for reactants during chemical processes. The oxygen mapping, which shows a similarly widespread distribution, confirms the integrity of the silica framework, suggesting that it remains intact while providing a stable environment for functionalization.

Sulfur is also mapped extensively, suggesting that benzothiazole groups containing sulfur atoms are evenly incorporated into the structure. This incorporation is crucial as sulfur can enhance catalytic activity through various mechanisms, such as coordination with metal centers or participation in redox reactions.

Chlorine appears more localized than other elements, indicating that its distribution may be influenced by specific interactions or bonding environments within the nanocatalyst. The presence of chlorine is significant because it can enhance reactivity and selectivity in catalytic applications.

Iron mapping reveals a distinct distribution pattern consistent with expectations from ferrocene integration. The presence of iron not only contributes to potential redox activity but indicates that ferrocene has been successfully incorporated into the nanostructure, which can facilitate electron transfer processes during catalysis.

Silicon mapping confirms that the silica backbone is uniformly distributed throughout the sample, providing structural stability essential for maintaining the integrity of the nanocatalyst under reaction conditions. Nitrogen mapping further supports this analysis by showing an even distribution across the material, affirming that benzothiazole groups containing nitrogen are effectively integrated into the composite.

Comparative analysis of these elemental distributions illustrates a well‐structured hybrid material where organic components are seamlessly integrated with inorganic silica. The uniformity observed in carbon, nitrogen, sulfur, and iron distributions suggests that effective functionalization strategies have been employed during synthesis. In contrast, variations in chlorine distribution may indicate specific roles or interactions within catalytic processes.

The elemental mapping is an invaluable tool for elucidating the composition and structural characteristics of SiO_2_@Benzothiazole‐Cl@Fc nanocatalyst. The findings underscore the successful integration of various elements and highlight their potential roles in enhancing catalytic performance. This detailed understanding lays a solid foundation for future investigations into optimizing this nanocatalyst for specific chemical transformations and applications in catalysis.

To showcase the efficiency of our proposed method for the preparation of substituted 1,3,5‐trisubstituted pyrazole derivatives, we synthesized product 4a as a representative example. We then conducted a comparative analysis of the results obtained using our method against those from previously documented approaches. As summarized in **Table** **3**, the findings highlight the distinct advantages of the proposed method. It utilizes milder and more sustainable reaction conditions and achieves significantly higher yields of the desired product compared to those reported in the literature. This underscores the potential of our approach in offering an efficient and environmentally friendly strategy for synthesizing valuable pyrazole derivatives.

**Table 3:** Comparison of the efficiency of this method with reported methods for the preparation of 1,3,5‐trisubstituted pyrazoles derivatives (**product 4a**).

Entry	Methodology	Ref.
1	PTSA (20 mol%), CH_2_Cl_2_, r.t., 8 h, 86%	[74]
2	A: n‐BuLi in THF 0 °C B: iodine/K_2_CO_3_ t‐BuOH, reflux, 2 h C: TEMPO (10 mol%), DIB, DCE, r.t., 4 h D: Fe(NO_3_).9H_2_O (10 mol%), TEMPO (10 mol%), r.t., 24 h	[75]
3	Cu(OTf)_2_ (20 mol %) CH_2_Cl_2_, reflux, 8 h, 89%	[76]
**4**	**SiO**_**2**_ **@Benzothiazole‐Cl@Fc (10 mg) CH**_**2**_ **Cl**_**2**_ **, reflux, 3 h, 95%**	**This work**

## 3 Conclusion

In this study, we successfully developed and characterized a novel silica nanospheres supporting a ferrocene‐containing IL (SiO_2_@Benzothiazole‐Cl@Fc) as a mild, efficient, and reusable heterogeneous catalyst for the A3 coupling reaction of aromatic hydrazides, aldehydes, and aromatic alkynes. The catalyst demonstrated excellent catalytic activity, affording 1,3,5‐trisubstituted pyrazoles in high yields under mild reaction conditions. The synergistic effect of the ferrocene moiety and the IL functionality, combined with the silica nanospheres’ high surface area and stability, contributed to the enhanced catalytic performance. Furthermore, the catalyst exhibited remarkable reusability, maintaining its activity and structural integrity over multiple reaction cycles, which underscores its potential for sustainable and green chemistry applications. This work provides an efficient approach for synthesizing biologically relevant pyrazole derivatives and highlights the versatility of functionalized silica‐based materials in heterogeneous catalysis. Future studies could explore the scope of this catalyst in other MCRs and its potential applications in industrial‐scale processes. The development of this heterogeneous catalyst has significant implications for the synthesis of pyrazole derivatives, which are of great interest in pharmaceutical and materials science applications. Furthermore, this study highlights the potential of ferrocene‐containing ILs as a new class of catalysts for organic synthesis. It paves the way for the design of novel heterogeneous catalysts for a wide range of chemical transformations.

## 4 Experimental Section

4.1

4.1.1

The reagents and chemical raw materials used in this research were meticulously sourced from Fluka and Merck and utilized as received without further purification. A Bruker AVANCE instrument, renowned for its precision, was employed to acquire ^1^H and ^13^C NMR spectral data, functioning at frequencies of 500 and 125 MHz, respectively. TMS and DMSO‐d_6_ served as internal standards and solvents. TEM images were obtained using a Philips transmission electron microscope, known for its high‐resolution imaging. Powder XRD data were collected with a Rigaku D‐max C III X‐ray diffractometer, utilizing Cu Kα radiation (λ = 1.54 Å). Melting points were recorded using an Electrothermal 9100 device. The microscopic morphology of the catalyst was examined using a scanning electron microscope (SEM, Philips, XL‐30) equipped with an EDX detector.

##### Synthesis of SiO_2_@propylchloride

Monodisperse silica nanospheres (SiO_2_) were synthesized via Stöber's method,^[^ ^73^ ^]^ a process known for its precision and accuracy. This method involves the hydrolysis of TEOS in the presence of ammonium hydroxide and ethanol. In a typical synthesis, 1 mL of TEOS was mixed with 10 mL of ethanol, followed by 10 mL of 25% ammonium hydroxide and an additional 10 mL of ethanol. The resulting reaction mixture was subjected to sonication. After 60 min, a white suspension of silica formed. The mixture was then centrifuged to isolate the silica nanospheres, which were washed with water and ethanol.

To functionalize the silica nanospheres with propyl chloride, a careful and thorough process was followed. 1 mL of 3‐chloropropyltriethoxysilane was added to a vigorously stirred dispersion of SiO_2_ in 50 mL of ethanol. The reaction was refluxed for 24 h, and the resulting propyl chloride‐functionalized silica nanospheres (SiO_2_@propyl chloride) were separated by centrifugation. They were then re‐dispersed in 50 mL of ethanol, and this washing process was repeated three times to ensure thorough functionalization.

##### For the Synthesis of 1‐(4‐Ferrocenylbutyl)Thiazole)

A suspension of 60% sodium hydride (NaH) (0.80 g, 20 mmol) in dry tetrahydrofuran (THF) (100 mL) was prepared, and thiazole (1.70 g, 20 mmol) was subsequently added. The mixture was stirred for 1 h at 0 °C, after which 4‐chlorobutylferrocene (1.38 g, 5 mmol) was introduced, and the reaction mixture was refluxed for 12 h. Upon completion of the reflux, the mixture was cooled to 0 °C, and any excess NaH was quenched with a small amount of water. The mixture was then transferred into water (50 mL) and extracted with dichloromethane (CH_2_Cl_2_). The combined organic extracts were dried over magnesium sulfate (MgSO_4_), and the solvent was evaporated under reduced pressure. The residue was purified via silica gel column chromatography using a solvent mixture of n‐hexane/ethyl acetate (9:1 v/v), yielding 1‐(4‐ferrocenylbutyl)‐1H‐imidazole as a brown viscous oil.

##### The Synthesis of SiO_2_@Benzothiazole‐Cl@Fc

The synthesis of SiO_2_@Benzothiazole‐Cl@Fc involved mixing SiO_2_@propyl chloride (B) (1.0 g) with 1‐N‐ferrocenylbutylimidazole (C) (0.92 g, 3 mmol) in 10 mL of toluene. The mixture was stirred at 80 °C for 72 h, after which the resultant product (SiO_2_@Benzothiazole‐Cl@Fc) (D) was filtered, washed with toluene (3 × 20 mL) and CH_2_Cl_2_ (3 × 20 mL), and then dried under vacuum at 50 °C for 48 h.

##### General Procedure for Synthesizing 1,3,5‐Trisubstituted Pyrazole Derivatives Using a SiO_2_@Benzothiazole‐Cl@Fc Nanocatalyst

In a beaker, a reaction mixture consisting of alkynes (1 mmol), an aromatic aldehyde (1 mmol), substituted phenylhydrazine (1.1 mmol), and 10 mg of the SiO_2_@Benzothiazole‐Cl@Fc nanocatalyst was prepared by adding 3 mL of dichloromethane. The reaction was carried out under reflux conditions, and the progress was monitored using thin‐layer chromatography (TLC) with an n‐hexane/ethyl acetate (1:3) eluent. Once the reaction was completed, the mixture was allowed to cool at room temperature for 10 min. The solid product was then filtered out, and the residue was dissolved in 3 mL of hot ethanol. The catalyst was recovered for reuse through centrifugation and subsequently washed with ethanol and dried in an oven. To isolate the pure pyrazole product, the ethanol solution was allowed to cool to room temperature, diluted with 1 mL of water, and left to crystallize. This process ensured the efficient separation and purification of the desired product while facilitating the reuse of the nanocatalyst.

##### NMR Information for 1,3,5‐Trisubstituted Pyrazoles: 1,3,5‐Triphenyl‐1H‐Pyrazole (4a)


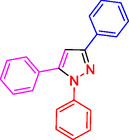


^1^HNMR (400 MHz, CDCl_3_): 7.88 (d, J = 7.7 Hz, 2 H), 7.75–7.70 (m, 4H), 7.68–7.61(m, 5H), 7.47–7.43 (m, 4H), 7.18 (s, 1H) ppm; ^13^C (100 MHz, CDCl_3_): 154.3, 148.0, 143.1, 137.6, 130.9, 129.0, 128.7, 127.3, 126.5, 125.4, 105.9 ppm; Mp 142–144 °C.


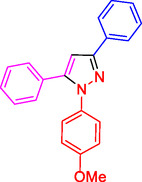


##### NMR Information for 1,3,5‐Trisubstituted Pyrazoles: 1‐(4‐Methoxyphenyl)‐3,5‐Diphenyl‐1H‐Pyrazole (4b)

^1^HNMR (400 MHz, CDCl_3_): 8.03 (d, J = 7.8 Hz, 2H), 7.95 (d, J = 8.0 Hz, 2 H), 7.87–7.81 (m, 4H), 7.77‐7.68 (m, 4H), 7.58 (d, J = 7.4 Hz, 2H), 7.13 (s, 1H), 3.83 (s, 3 H) ppm;^13^C (100 MHz, CDCl_3_): 159.7, 152.0, 147.6, 139.8, 137.9, 129.6, 128.2, 127.3, 126.0, 125.4, 124.5, 122.7, 114.8, 105.4, 54.6 ppm; Mp 117–119 °C.


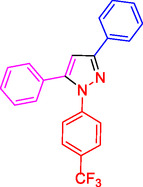


##### NMR Information for 1,3,5‐Trisubstituted Pyrazoles: 3,5‐Diphenyl‐1‐(4‐(trifluoromethyl)phenyl)‐1H‐Pyrazole (4c)

^1^HNMR (400 MHz, CDCl_3_): 7.82 (d, J = 8.6 Hz, 2H), 7.74 (d, J = 8.4 Hz, 2H), 7.65–7.61 (m, 4H), 7.59–7.51 (m, 4H), 7.43 (d, J = 7.7 Hz, 2H), 7.09 (s, 1H) ppm;^13^C (100 MHz, CDCl_3_): 154.7, 145.2, 143.1, 132.4, 130.7, 129.1, 127.7, 126.5, 122.3, 121.9, 106.5 ppm; Mp 114‐116 °C.


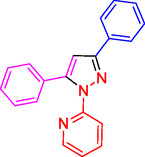


##### NMR Information for 1,3,5‐Trisubstituted Pyrazoles: 2‐(3,5‐Diphenyl‐1H‐Pyrazol‐1‐yl)pyridine (4d)

^1^HNMR (400 MHz, CDCl_3_): 8.43 (d, J = 7.4 Hz, 1H), 7.98 (d, J = 8.0 Hz, 2H), 7.84 (dd, J = 8.8, 3.7 Hz, 2H), 7.76–7.71 (m, 4H), 7.64–7.60 (m, 1H), 7.37 (s, 1H), 7.07–7.02 (m, 4H) ppm;^13^C (100 MHz, CDCl_3_): 152.8, 148.3, 142.7, 140.1, 132.4, 129.0, 128.6, 126.4, 125.1, 120.8, 112.5, 103.6 ppm; Mp 115–117 °C.


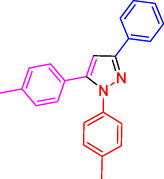


##### NMR Information for 1,3,5‐Trisubstituted Pyrazoles: 3‐Phenyl‐1,5‐di‐p‐Tolyl‐1H‐Pyrazole (4e)

^1^HNMR (400 MHz, CDCl_3_): 8.04 (d, J = 6.8 Hz, 2H), 7.58 (dd, J = 10.1, 4.5 Hz, 4H), 7.48–7.41 (m, 3H), 7.32 (d, J = 8.0 Hz, 2 H), 7.26 (d, J = 8.6 Hz, 2H), 7.12 (s, 1H), 2.47 (s, 3H), 2.32 (s, 3H) ppm;^13^C (100 MHz, CDCl_3_): 151.8, 142.3, 135.7, 129.0, 128.5, 127.6, 126.8, 125.3, 124.1, 105.4, 20.9 ppm; Mp 93–95 °C.


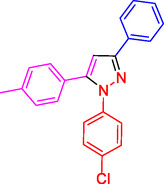


##### NMR Information for 1,3,5‐Trisubstituted Pyrazoles: 1‐(4‐Chlorophenyl)‐3‐Phenyl‐5‐(p‐Tolyl)‐1H‐Pyrazole (4f)

^1^HNMR (400 MHz, CDCl_3_): 8.03 (d, J = 7.6 Hz, 2H), 7.66 (d, J = 7.8 Hz, 2H), 7.58–7.51 (m, 3H), 7.42 (d, J = 7.6 Hz, 2H), 7.13 (dd, J = 9.4, 3.2 Hz, 2H), 7.05 (s, 1H), 2.36 (s, 3H) ppm;^13^C (100 MHz, CDCl_3_): 153.4, 146.1, 140.9, 134.2, 132.6, 131.8, 130.7, 129.0, 128.1, 125.4, 124.9, 108.1, 21.7 ppm; Mp 128–130 °C.


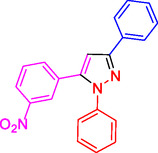


##### NMR Information for 1,3,5‐Trisubstituted Pyrazoles: 5‐(3‐Nitrophenyl)‐1,3‐Diphenyl‐1H‐Pyrazole (4g)

^1^HNMR (400 MHz, CDCl_3_): 8.54 (s, 1H), 8.32 (d, J = 7.6 Hz, 1H), 8.03 (d, J = 8.0 Hz, 1H), 7.96–91 (m, 1H), 7.87 (d, J = 7.9 Hz, 2H), 7.65–7.61 (m, 4H), 7.43–7.38 (m, 24H), 7.03 (s, 1H) ppm;^13^C (100 MHz, CDCl_3_): 153.0, 140.7, 136.5, 131.2, 129.7, 128.4, 127.8, 126.4, 125.3, 124.2, 107.6 ppm; Mp 132–135 °C.


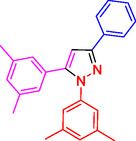


##### NMR Information for 1,3,5‐Trisubstituted Pyrazoles: 1,5‐bis(3,5‐Dimethylphenyl)‐3‐Phenyl‐1H‐Pyrazole (4h)

^1^HNMR (400 MHz, CDCl_3_): 8.05 (d, J = 8.0 Hz, 2H), 7.73 (s, 2H), 7.65 (s, 2H), 7.58–7.48 (m, 3H), 7.34 (s, 1H), 7.29 (s, 1H), 7.01 (s, 1H), 2.36 (s, 12H) ppm;^13^C (100 MHz, CDCl_3_): 153.0, 142.6, 138.7, 134.2, 130.7, 129.4, 128.6, 127.2, 126.3, 125.4, 124.0, 123.2, 119.8, 107.4, 20.8 ppm.


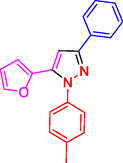


##### NMR Information for 1,3,5‐Trisubstituted Pyrazoles: 5‐(furan‐2‐yl)‐3‐Phenyl‐1‐(p‐Tolyl)‐1H‐Pyrazole (4i)

^1^HNMR (400 MHz, CDCl_3_): 8.07 (d, J = 9.0 Hz, 3H), 7.63 (d, J = 7.5 Hz, 1H), 7.55–7.48 (m, 6H), 7.38 (d, J = 8.6 Hz, 2H), 7.25 (s, 1H), 7.07‐7.02 (m,1H), 2.47 (s, 3H) ppm;^13^C (100 MHz, CDCl_3_): 152.3, 145.4, 134.7, 131.2, 130.8, 130.0, 129.5, 128.3, 127.4, 124.0, 122.1, 108.9, 105.5, 21.4 ppm.


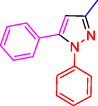


##### NMR Information for 1,3,5‐Trisubstituted Pyrazoles: 3‐Methyl‐1,5‐Diphenyl‐1H‐Pyrazole (4j)

1HNMR (400 MHz, CDCl_3_): 7.84–7.80 (m, 4H), 7.66–7.61 (m, 1H), 7.43–7.39 (m, 1H), 7.06–7.02 (m, 4H), 6.53 (s, 1H), 2.33 (s, 3H) ppm;^13^C (100 MHz, CDCl_3_): 152.7, 145.1, 140.3, 133.0, 129.8, 128.2, 127.5, 126.3, 124.8, 107.4, 14.3 ppm; Mp 49–51 °C.


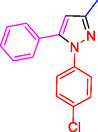


##### NMR Information for 1,3,5‐Trisubstituted Pyrazoles: 1‐(4‐Chlorophenyl)‐3‐methyl‐5‐phenyl‐1*H*‐pyrazole (4k)

^1^HNMR (400 MHz, CDCl_3_): 7.78 (d, J = 8.6 Hz, 2H), 7.63 (d, J = 7.6 Hz, 2H), 7.47–7.43 (m, 4H), 7.33–7.29 (m, 1H), 6.52 (s, 1H), 2.38 (s, 3H) ppm;^13^C (100 MHz, CDCl_3_): 152.1, 141.9, 133.2, 132.0, 130.7, 129.6, 128.4, 127.3, 125.2, 105.4, 17.6 ppm; Mp 70–72 °C.


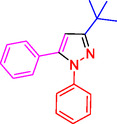


##### NMR Information for 1,3,5‐Trisubstituted Pyrazoles: 3‐(Tert‐Butyl)‐1,5‐Diphenyl‐1H‐Pyrazole (4L)

^1^HNMR (400 MHz, CDCl_3_): 7.76–7.71 (m, 4H), 7.63–7.60 (m, 1H), 7.48–7.43 (m, 1H), 7.24–7.20 (m, 4H), 6.52 (s, 1H), 1.27 (s, 9H) ppm;^13^C (100 MHz, CDCl_3_): 167.1, 144.3, 135.9, 129.5, 128.7, 127.4, 126.1, 125.6, 106.1, 31.2, 28.9 ppm; Mp 107–109 °C.


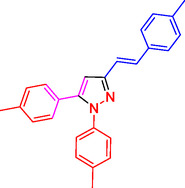


##### NMR Information for 1,3,5‐Trisubstituted Pyrazoles: (E)‐3‐(4‐Methylstyryl)‐1,5‐di‐p‐Tolyl‐1H‐Pyrazole (4m)

^1^HNMR (400 MHz, CDCl_3_): 7.76 (dd, J = 9.2, 5.3 Hz, 4H), 7.53 (dd, J = 8.9, 3.5 Hz, 2H), 7.44 (d, J = 8.0 Hz, 2H), 7.32 (d, J = 8.6 Hz, 4H), 7.07 (s, 1H), 6.89 (s, 2H), 2.42 (s, 6H), 2.37 (s, 3H) ppm;^13^C (100 MHz, CDCl_3_): 158.0, 143.2, 134.5, 130.9, 129.7, 128.2, 127.4, 126.3, 110.7, 108.6, 22.5 ppm; Mp 173–175 °C.


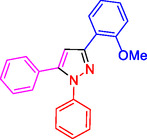


##### NMR Information for 1,3,5‐Trisubstituted Pyrazoles: 3‐(2‐Methoxyphenyl)‐1,5‐Diphenyl‐1H‐Pyrazole (4n)

^1^HNMR (400 MHz, CDCl_3_): 8.37 (d, J = 7.6 Hz, 2H), 7.84–7.80 (m, 3H), 7.46 (t, J = 8.4 Hz, 4H), 7.38–7.33 (m, 1H), 7.28–7.20 (m, 2H), 7.12 (d, J = 7.7 Hz, 2H), 7.03 (s, 1H), 3.83 (s, 3H) ppm;^13^C (100 MHz, CDCl_3_): 158.6, 154.3, 146.0, 140.1, 131.3, 130.7, 129.8, 128.5, 127.2, 126.5, 125.1, 124.3, 111.9, 104.3, 55.8 ppm; Mp 89–91 °C.


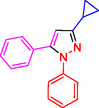


##### NMR Information for 1,3,5‐Trisubstituted Pyrazoles: 3‐cyclopropyl‐1,5‐diphenyl‐1*H*‐pyrazole (4o)

_1_HNMR (400 MHz, CDCl_3_): 7.75–7.70 (m, 4H), 7.64‐7.61 (m, 2H), 7.56–7.47 (m, 4H), 6.53 (s, 1H), 2.38–2.33 (m, 1H), 1.29–1.25 (m, 2H), 1.06–1.01 (m, 2H) ppm;^13^C (100 MHz, CDCl_3_): 170.6, 140.0, 132.4, 129.8, 128.1, 127.3, 125.3, 124.0, 107.1, 9.6, 8.0 ppm.


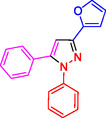


##### NMR Information for 1,3,5‐Trisubstituted Pyrazoles: 3‐(furan‐2‐yl)‐1,5‐Diphenyl‐1H‐Pyrazole (4p)

^1^HNMR (400 MHz, CDCl_3_): 8.02 (d, J = 7.6 Hz, 1H), 7.67–7.60 (m, 5H), 7.54–7.52 (m, 2H), 7.27 (s, 1H), 7.15–7.08 (m, 4H), 6.88–6.82 (m, 1H) ppm;^13^C (100 MHz, CDCl_3_): 158.0, 140.7, 133.2, 132.7, 130.4, 129.6, 128.2, 127.5, 126.0, 112.0, 104.3 ppm; Mp 128–130 °C.


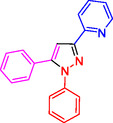


##### NMR Information for 1,3,5‐Trisubstituted Pyrazoles: 2‐(1,5‐Diphenyl‐1H‐Pyrazol‐3‐yl)pyridine (4q)

^1^HNMR (400 MHz, CDCl_3_): 7.76 (dd, J = 9.2, 5.3 Hz, 4H), 7.53 (dd, J = 8.9, 3.5 Hz, 2H), 7.44 (d, J = 8.0 Hz, 2H), 7.32 (d, J = 8.6 Hz, 4H), 7.07 (s, 1H), 6.89 (s, 2H), 2.42 (s, 6H), 2.37 (s, 3H) ppm;^13^C (100 MHz, CDCl_3_): 154.7, 148.7, 142.1, 141.9, 140.1, 139.7, 132.4, 128.3, 127.4, 127.0, 126.5, 126.1, 125.4, 124.3, 103.7 ppm; Mp 111–113 °C.


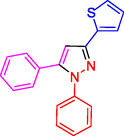


##### NMR Information for 1,3,5‐Trisubstituted Pyrazoles: 1,5‐Diphenyl‐3‐(thiophen‐2‐yl)‐1H‐pyrazole (4r)

^1^HNMR (400 MHz, CDCl_3_): 7.84–7.80 (m, 4H), 7.48–43 (m, 3H), 7.37 (s, 1H), 7.29 (t, J = 8.0 Hz, 1H), 7.18–7.12 (m, 4H) ppm;^13^C (100 MHz, CDCl_3_): 142.8, 140.9, 138.1, 131.8, 130.7, 129.5, 128.3, 127.6, 126.1, 125.4, 105.8 ppm; Mp 85–87 °C.

## Conflict of Interest

The authors declare no conflict of interest.

## Author Contributions

**Ramin Javahershenas**: investigation (equal); methodology (equal); project administration (lead); writing—original draft (equal); writing—review and editing (equal). **Fadhil Faez Sead**: investigation (equal); methodology (equal); writing—original draft (equal); writing—review and editing (equal). **Vicky Jain**: investigation (equal); methodology (equal); writing—original draft (equal); writing—review and editing (equal). **Roopashree R**: investigation (equal); methodology (equal); writing—original draft (equal); writing—review and editing (equal); **Aditya Kashyap**: investigation (equal); methodology (equal); writing—original draft (equal); writing—review and editing (equal); **Suman Saini**: investigation (equal); methodology (equal). **Girish Chandra Sharma**: investigation (equal); methodology (equal). **Pushpa Negi Bhakuni**: writing—original draft (equal); writing—review and editing (equal). **Mosstafa Kazemi**: methodology (equal); project administration (equal); writing—original draft (equal); writing—review and editing (equal).

## Data Availability

All data generated or analyzed during this study are included in this published article and its supplementary information files.
